# Acute self-myofascial release modulates cardiac autonomic function and hemodynamic parameters at rest and reduces cardiovascular stress reaction

**DOI:** 10.1007/s00421-023-05382-2

**Published:** 2023-12-29

**Authors:** Sascha Ketelhut, Livia Oechslin, Cäcilia Zehnder, Claudia Kubica, Claudio R. Nigg

**Affiliations:** Institute of Sports Science, University of Bern, Bremgartenstrasse 145, 3013 Bern, Switzerland

**Keywords:** Foam roller, Heart rate variability, Blood pressure, Pain perception, Cold pressor test

## Abstract

**Purpose:**

Self-myofascial release (SMR) is a form of self-massage aiming to release tension, improve blood flow, and alleviate muscle soreness. This study aimed to determine whether a single session of SMR could impact cardiovascular parameters at rest and during a cold pressor test (CPT).

**Methods:**

Twenty male participants (aged 26 ± 2 years) underwent a 20-min SMR and a 20-min seated control condition (CON) on two separate test days in a randomized order. Peripheral and central blood pressure (BP), total peripheral resistance (TPR), pulse wave velocity (PWV), heart rate (HR), root mean square of successive RR interval differences (RMSSD), and the quotient of low-frequency power and high-frequency power (LF/HF) were measured both at rest and during a CPT before (t0), 2 min (t1), and 20 min (t2) after the SMR and CON.

**Results:**

Time × condition interactions could be detected for peripheral and central diastolic BP, TPR, HR, and RMSSD. Following the SMR, peripheral diastolic BP, central diastolic BP, TPR, and RMSSD were reduced, while HR was increased compared to the CON. Regarding the CPT time × condition interactions could be detected for peripheral, and central diastolic BP, with lower values after SMR.

**Conclusion:**

The results of the present study suggest that a single bout of SMR confers favorable cardiovascular benefits in healthy normotensive individuals. Furthermore, SMR can attenuate the hemodynamic reactivity to a stress test. Future research should address whether regular SMR leads to chronic adaptations similar to regular, moderate aerobic exercise, massage therapy, and static stretching.

## Introduction

Despite the availability of established prevention and treatment approaches, cardiovascular diseases have remained the leading cause of disease burden for decades (Roth et al. 2020). In addition to pharmacotherapy and lifestyle changes, stretching exercises and manual therapies such as massage therapy are gaining popularity as potential treatment approaches for cardiovascular health.

Research has shown that massage therapy lowers systolic and diastolic blood pressure in individuals with hypertension or prehypertension (Liao et al. 2016). Acute stretching exercises have also been found to trigger cardiovascular responses, which, if performed regularly, can result in improved endothelial function and reduced arterial stiffness and blood pressure (Nishiwaki et al. 2015; Kruse and Scheuermann 2017). It has been assumed that the mechanical stress applied to vessels during stretching exercises or massage therapy can trigger relevant hemodynamic adaptations (Kuebler et al. 2003; Lu and Kassab 2015). Among manual therapy techniques, self-myofascial release (SMR) has recently gained popularity in rehabilitation and fitness settings. SMR is a type of myofascial release, which includes manual therapy techniques that induce pressure to the muscle and fascia and is performed by the individuals themselves, often involving a tool (Beardsley and Škarabot 2015). SMR has been found to increase the range of motion, reduce perceived pain, accelerate recovery, and enhance exercise performance (Beardsley and Škarabot 2015; Cheatham and Stull 2019; Wiewelhove et al. 2019).

As SMR combines mechanical compression and stretching impulses similar to massage therapy and stretching exercises, it is plausible that SMR can also trigger hemodynamic responses. Emerging evidence has demonstrated the positive effects of SMR on arterial function, blood pressure, and modulation of autonomic nervous system activity at rest (Okamoto et al. 2013; Chan et al. 2015; Lastova et al. 2018; Ketelhut et al. 2020).

Apart from assessing hemodynamic parameters at rest, stress related hyperreactivity of the cardiovascular system has been identified as a relevant risk factor for the development of hypertension and cardiovascular diseases (Zhao et al. 2015). It has been suggested that frequent fluctuations in sympathetic activity and thus, changes in blood pressure, due to stress, can directly affect the vascular system, causing damage and affecting arterial compliance, which in long term could result in hypertension (Folkow 1993). As both chronic and acute stress are inherent elements of everyday life, attenuating cardiovascular responses to stress can play a crucial role in preventing cardiovascular diseases.

The cold pressor test is a validated method for evaluating cardiovascular reactivity (Pouwels et al. 2019). Previous studies have demonstrated that acute exercise results in positive modulations of hemodynamic responses to the cold pressor test (Milatz et al. 2015; Ketelhut et al. 2016). Considering the potential of SMR to positively affect the cardiovascular system and the analgesic effects mediated by peripheral, spinal, or supraspinal mechanisms (Bialosky et al. 2009), it is plausible that SMR modulates hemodynamic reactivity during stress exposure. To the best of our knowledge, no studies have assessed the effects of SMR on hemodynamic reactivity to a standardized stress test.

This study aims to evaluate the immediate effects of acute SMR on peripheral and central blood pressure, heart rate, heart rate variability (HRV), total peripheral resistance (TPR), and pulse wave velocity (PWV). Furthermore, it will investigate whether SMR can influence hemodynamic reactivity and perceived pain during a cold pressor test.

We hypothesize that the SMR session will have a positive impact on hemodynamic and HRV parameters at rest. In addition, we anticipate that SMR will reduce hemodynamic and cardiac autonomic reactivity and mitigate perceived pain during a cold pressor test.

## Methods

### Participants

The study was conducted on a group of healthy males who met the following inclusion criteria: (1) age 18 or older, (2) had no prior experience with SMR and foam rolling, (3) were not involved in sports that require a high level of mobility, (4) had no underlying medical conditions that could potentially compromise the safety of physical exercise, and (5) were not taking cardiovascular medications. Participants were recruited between January and February 2021 through personal contacts and social media platforms.

An a priori power analysis using G*Power (Version 3.1.2; Heinrich Heine Universität, Dusseldorf, Germany) was conducted. Assuming an effect size of 0.4 for pSBP (Ketelhut et al. 2020) with an alpha level of 0.05, 16 participants would be necessary for an appropriate power (0.8). Therefore, twenty normotensive young and healthy participants took part in the study. Prior to enrollment, the participants were provided with information regarding the study’s objectives and procedures, and written consent was obtained from each participant.

### Study design

A randomized crossover study design was implemented. All measurements were carried out in the physiology laboratory of the Institute of Sports Science at the University of Bern, ensuring a controlled environment. The participants attended the laboratory on 2 separate test days, with a washout period of at least 48 h and no longer than 7 days. The sessions were scheduled at the same times of the day. Prior to the measurements, participants were instructed to arrive at the lab at least 4 h after their last meal and to abstain from consuming caffeinated or alcoholic beverages, as well as nicotine, for 4 h. They were also advised to refrain from engaging in intense physical activity for a minimum of 24 h preceding each test day.

**Fig. 1:**
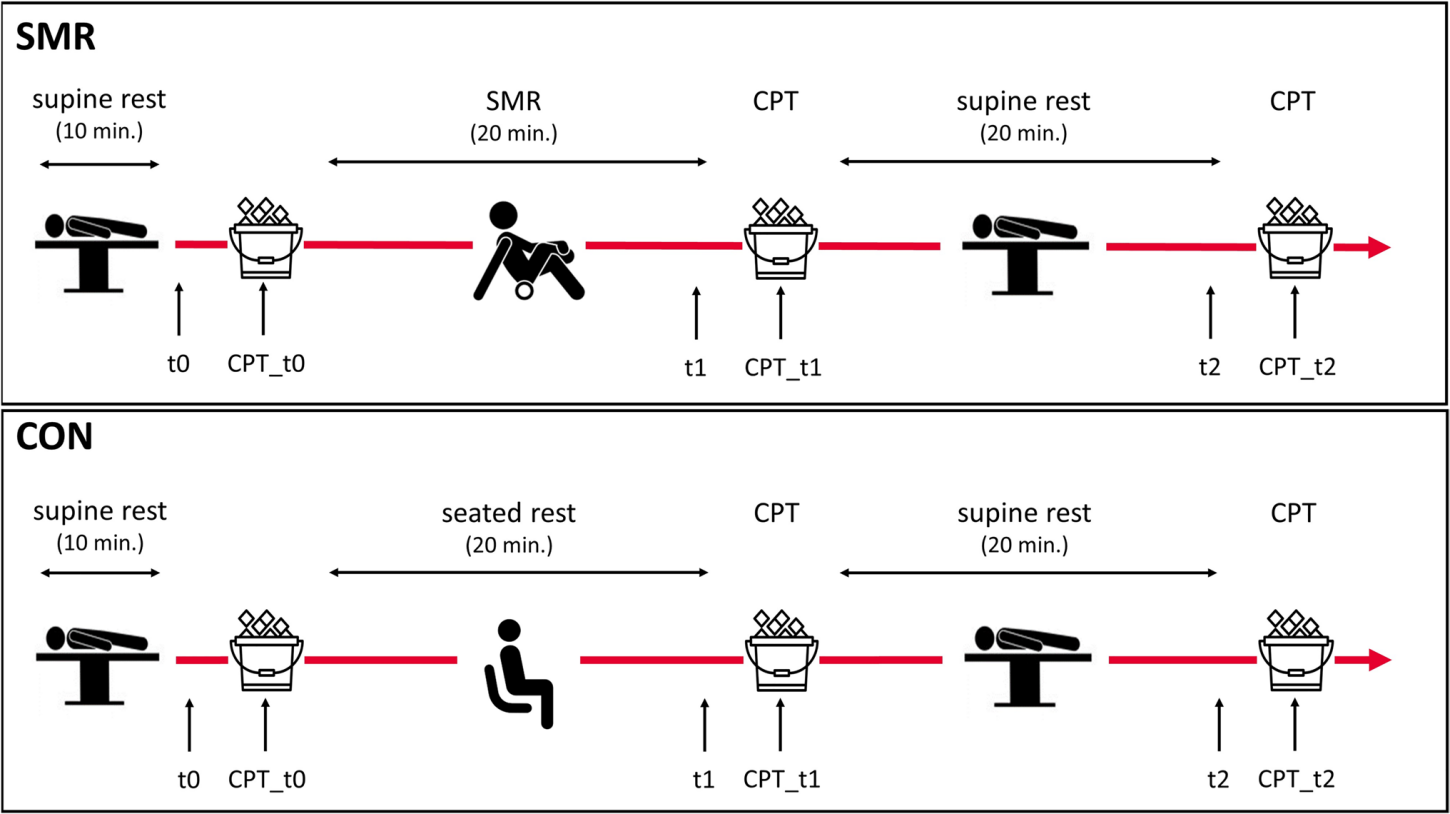
Study design. *SMR* self-myofascial release, *CON* control condition, *CPT* cold pressor test, *t0* rest, *t1* post SMR or seated rest, *t2* post supine rest

On each test day, hemodynamic parameters and cardiac autonomic function were evaluated at rest (t0) and during a cold pressor test (CPT_t0). Following this, participants either engaged in a 20-min SMR exercise or a 20-min seated rest (CON). The randomization was performed by the principal investigator using the random sequence generator software at www.random.org. After each condition, outcomes were assessed again at rest (t1) and during a cold pressor test 2 min after the condition (CPT_t1), as well as after a 20-min period of supine rest (t2, CPT_t2) (Fig. 1).

The measurements were conducted by trained study staff who utilized the same equipment and ensured a standardized procedure. The study was conducted in accordance with the Declaration of Helsinki and approved by the Ethical Commission of the Faculty of Human Sciences University of Bern (Nr. 2021-03-00003).

### Self-myofascial release

The participants performed a SMR exercise using a commercially available foam roller BLACKROLL^®^ standard (Blackroll AG, Bottighofen, Switzerland) with a diameter of 15 cm and a length of 30 cm. The protocol adhered to the recommendations of Behm et al. (2020) and specifically targeted the calf muscles, the outer thigh, the front thigh, the inner thighs, and the buttocks (Table 1). The procedure involved rolling the specific muscle groups back and forth across the foam roller, working the entire surface area. Each exercise comprised of two sets, with each set lasting 60 s, followed by a 60-s rest period between sets. Participants were instructed to maintain a steady and controlled rolling motion (10 × back and forth per minute), synchronizing their movements with the rhythm of a metronome. During rest periods, participants rolled the opposite extremity. The intensity of the exercise was regulated using body weight to apply pressure to the soft tissues during the rolling motion. The non-engaged legs and arms were used as needed to offset weight during the exercise. The participants were instructed to apply a pressure level that induced bearable pain, rating around 7–8 on a pain scale where 0 represented no pain at all and 10 indicated the maximum tolerable pain. An investigator provided continuous feedback throughout the exercise to ensure that participants maintained proper form and rhythm.

**Table 1:** Description of self-myofascial release exercises

Muscle area	Exercise description
Calf	The calf was positioned on the fascia roller, with one leg extended while the other leg was parallel to the roller on the floor for added stability. The participants supported themselves laterally with their hands. The roller was then moved from below the knee joint to just above the heel and rolled back again
Outer thigh	The participants positioned themselves in the lateral plank posture, with the lower outer side of the thigh resting on the fascia roller. The second leg was angled in front of the targeted leg to ensure stability. The rolling motion began slightly below the pelvis, smoothly gliding upward toward the region just above the knee joint, and then retraced the same path in a controlled manner
Front thigh	Participants moved into the forearm plank position, resting the front of their thigh on the foam roller. The second leg was positioned parallel to the roller on the floor for added stability. The exercise involved a controlled movement that spanned from below the pubic bone to just above the knee joint, and then returned to the starting position
Inner thigh	The participants moved into the forearm plank position and bent the corresponding leg. They positioned the inner side of their thigh on the fascia roller while keeping the other leg extended on the floor. With a controlled motion, the participants rolled the roller from below the pubic bone up to just above the knee joint and then returned to the starting position
Buttocks	The participants seated themselves with the corresponding side of their buttocks resting on the roller. They supported themselves by placing their hands either behind their body or behind the roller and slightly turned to the side that was being rolled. The foot of the side to be worked on is placed on the opposite knee

### Control condition

During the control (CON) condition, participants remained seated on a chair for 20 min. They were instructed to stay calm, recline slightly, ensure their feet were flat on the floor, and avoid falling asleep. The use of entertainment media was not allowed.

## Measurements

### Anthropometrics

Height, body mass, and body composition were assessed using a stadiometer and scale (BC-545 Innerscan, Tanita, Netherlands). Waist circumference was measured with a precision of 0.1 cm at the midpoint between the iliac crest and the lowest ribs. Body Mass Index (BMI) was calculated as weight in kilograms divided by the square of height in meters (kg/m^2^). The Waist-to-height Ratio (WHtR) was determined by dividing the waist circumference by the height (waist circumference/height).

### Hemodynamic measurements

The non-invasive measurement of hemodynamic parameters, including peripheral systolic blood pressure (pSBP), peripheral diastolic blood pressure (pDBP), central systolic blood pressure (cSBP), central diastolic blood pressure (cDBP), PWV, and TPR was carried out using the Mobil-O-Graph^®^ (PWA-Monitor, IEM, Stollberg, Germany), which is a validated device for hemodynamic assessment (Franssen and Imholz 2010). Following a 10-min period of rest in a supine position, three measurements were taken on the right upper arm using custom-fitted arm cuffs. To ensure that the heart and pressure cuff were at the same level, the arm was placed on an armrest during the measurements. For analysis, the average of the second and third readings was used.

### Cardiac autonomic function

To measure HRV, a heart rate monitor and chest strap (Polar RS800 CX^®^, Polar Electro OY, Kempele, Finland) were employed. After 10-min period of supine rest to establish a stable HRV signal, a 5.5-min measurement was taken. The RR intervals were recorded at a sampling rate of 1000 Hz (Task Force of the European Society of Cardiology the North American Society of Pacing Electrophysiology 1996). Participants were instructed to breathe normally, refrain from speaking, and maintain calm during the measurement. Data collected during the final five minutes of the measurement were subjected to analysis. For processing raw data the software “Kubios HRV” version 2.1 (Biosignal Analysis and Medical Imaging Group, Department of Physics, University of Kuopio, Kuopio, Finland), was used. The default settings of Kubios preprocessing were employed, including the RR detrending method, which was set to “Smoothen priors” (Lambda = 500) (Tarvainen et al. 2014). Only data with an error ratio below 5% were considered for analysis (Task Force of the European Society of Cardiology the North American Society of Pacing Electrophysiology 1996). The root mean square of successive differences between normal heartbeats (RMSSD in ms), the quotient of low-frequency power and high-frequency power (LF/HF).

### Cold pressure test

The cold pressor test is a widely recognized and validated method for assessing cardiovascular reactivity, as evidenced by numerous studies (Pouwels et al. 2019). Trained study personnel conducted the cold pressor test using a standardized protocol. The participants were instructed to immerse their right hand in cold water (5.0 ± 0.1 °C) for a duration of 2 minutes, during which blood pressure, PWV, heart rate, and HRV parameters were measured. The participants’ pain level during the test was evaluated using a numerical rating scale ranging from 0 to 10, with 0 indicating no pain and 10 indicating the most intense pain imaginable.

### Data analysis

Data were analyzed using SPSS version 26.0 (IBM Corp., Armonk, NY, USA). Data were examined for normality with the Shapiro–Wilk test. A series of time (t0, t1, t2) × condition (SMR vs. CON) repeated-measures analysis of variance (ANOVA) were calculated to determine interactions for parameters at rest and during the cold pressor test. Significant interactions or main effects were analyzed using a Bonferroni post hoc test. Partial eta-squared (*η*^2^_*p*_) values were calculated to estimate the effect sizes (small effect: *η*^2^_*p*_ = 0.014, medium effect: *η*^2^_*p*_ = 0.06, large effect: *η*^2^_*p*_ = 0.14) for the interactions. Statistical significance was set at *p* < 0.05.

## Results

No adverse events occurred during the examination sessions in any of the participants. All 20 participants completed both test days. The characteristics of the participants are presented in Table 2.

**Table 2:** Participants’ characteristics

Outcomes	*M* ± *SD*	Range
Age (yrs)	26 ± 2	21–32
Body mass (kg)	76.1 ± 7.2	62.5–91.6
Height (cm)	182.6 ± 6.9	171–196
Waist-to-height ratio	0.45 ± 0.04	0.39–0.55
Body fat (%)	17 ± 5	10.8–30.7
Body Mass Index (kg/m^2^)	23 ± 2.3	19.5–28.8
Systolic blood pressure (mmHg)	121 ± 8	105.5–136.5
Diastolic blood pressure (mmHg)	71 ± 6	57–83

*M* Mean, *SD* standard deviation. *n* = 20

According to the Body Mass Index, four participants were classified as overweight (> 25 kg/m^2^). Based on the waist-to-height ratio, two of the participants included showed values in the overweight range (> 0.5). According to pSBP, none of the participants could be classified as hypertensive (Williams et al. 2018). Three of the participants presented a high normal pSBP. According to the pDBP, all participants were classified as normotensive.

### Hemodynamic parameters

**Fig. 2:**
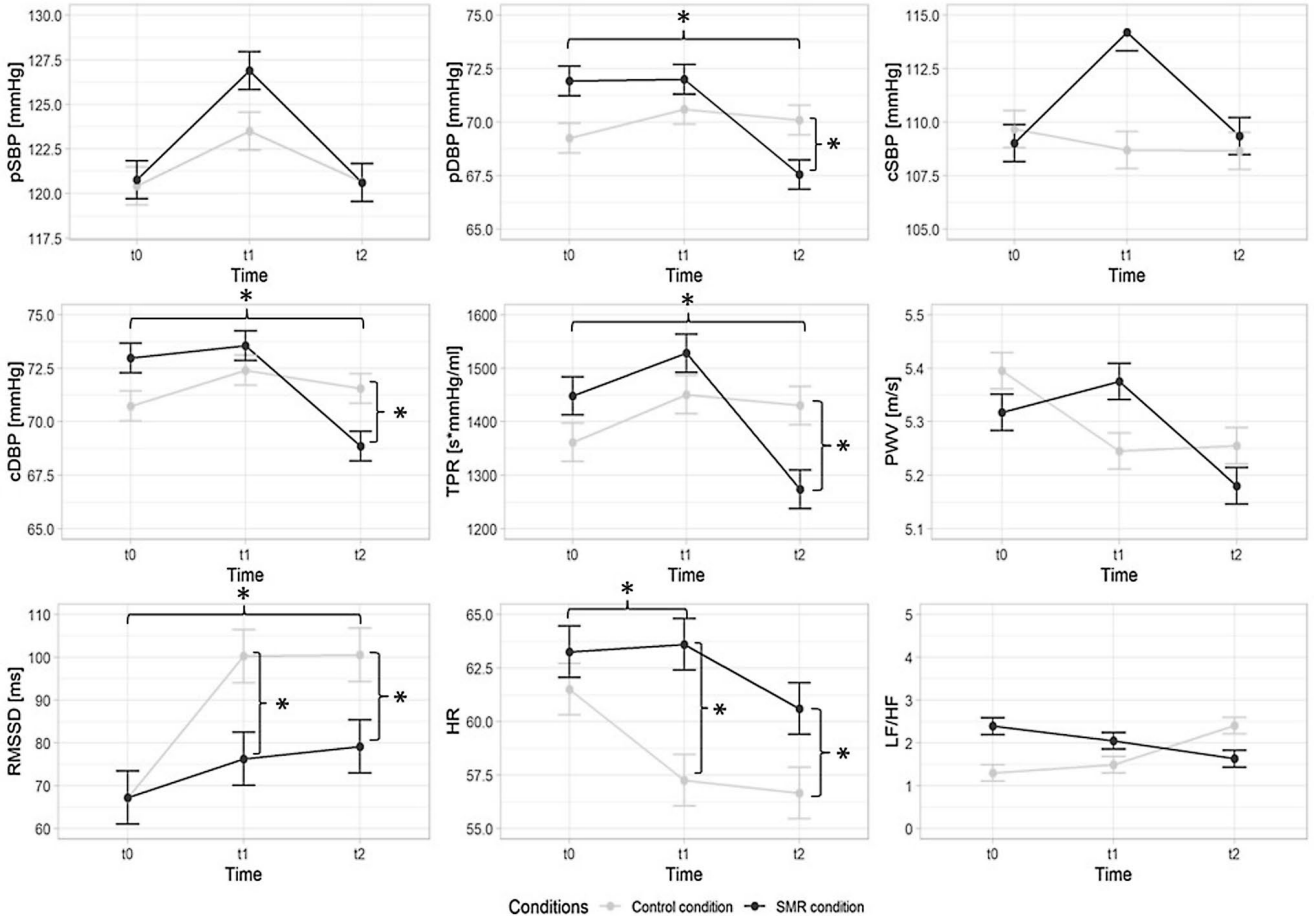
Outcomes during resting measurements at baseline (t0), 2 min (t1), and 20 min (t2) after the control condition and the self-myofascial release (SMR) condition. Error bars represent standard errors; *pSBP* peripheral systolic blood pressure, *pDBP* peripheral diastolic blood pressure, *cSBP* central systolic blood pressure, *cDBP* central diastolic blood pressure, *TPR* total peripheral resistance, *PWV* pulse wave velocity, *RMSSD* root mean square of successive differences between normal heartbeats, *HR* heart rate, *LF/HF* low-frequency power and high-frequency power quotient

A time × condition interaction was observed for pDBP (*F*(2,38) = 14.341, *p* < 0.001, *η*^*2*^_*p*_ = 0.430) (Fig. 2). The SMR session resulted in a more pronounced reduction from t0 to t2 compared to the CON condition (*F*(2,38) = 34.669,* p* < 0.001, *η*^*2*^_*p*_ = 0.646), resulting in a significantly lower pDBP at t2 (*p* = 0.027). Similarly, a time × condition interaction was observed for cDBP (*F*(2,38) = 9.790, *p* < 0.001, *η*^*2*^_*p*_ = 0.340), with a stronger reduction from t0 to t2 following the SMR condition (*F*(2,38) = 21.911, *p* < 0.001, *η*^*2*^_*p*_ = 0.536). This resulted in a significantly lower cDBP at t2 (*p* = 0.030). The TPR demonstrated a time × condition interaction (*F*(2,38) = 5.525, *p* = 0.008, *η*^*2*^_*p*_ = 0.225). Following the SMR condition there was a stronger reduction from t0 to t2 (F(2,38) = 6.817, *p* = 0.017, *η*^*2*^_*p*_ = 0.264), resulting in a significantly lower TPR at t2 (*p* = 0.024). No time × condition interaction effects could be observed for pSBP, cSBP, PWV, LF/HF (*ps* > 0.05, *η*^*2*^_*p*_ = 0.039–0.153).

### Cardiac autonomic function

A time × condition interaction was found for HR (*F*(2,38) = 3.422, *p* = 0.043, *η*^*2*^_*p*_ = 0.153) (Fig. 2). The CON condition resulted in a stronger reduction from t0 to t1 (*F*(2,38) = 6.817,* p* = 0.017, *η*^*2*^_*p*_ = 0.264), leading to a lower HR at t1 (*p* < 0.001) and t2 (*p* = 0.007). The analysis of RMSSD also revealed a time × condition interaction (*F*(2,38) = 3.901, *p* = 0.047, *η*^*2*^_*p*_ = 0.170). A more substantial increase in RMSSD from t0 to t1 was detected following the CON condition (*F*(2,38) = 4.517, *p* = 0.047, *η*^*2*^_*p*_ = 0.192), leading to a significantly lower RMSSD after the CON condition at t1 (*p* = 0.006).

### Stress response

**Fig. 3:**
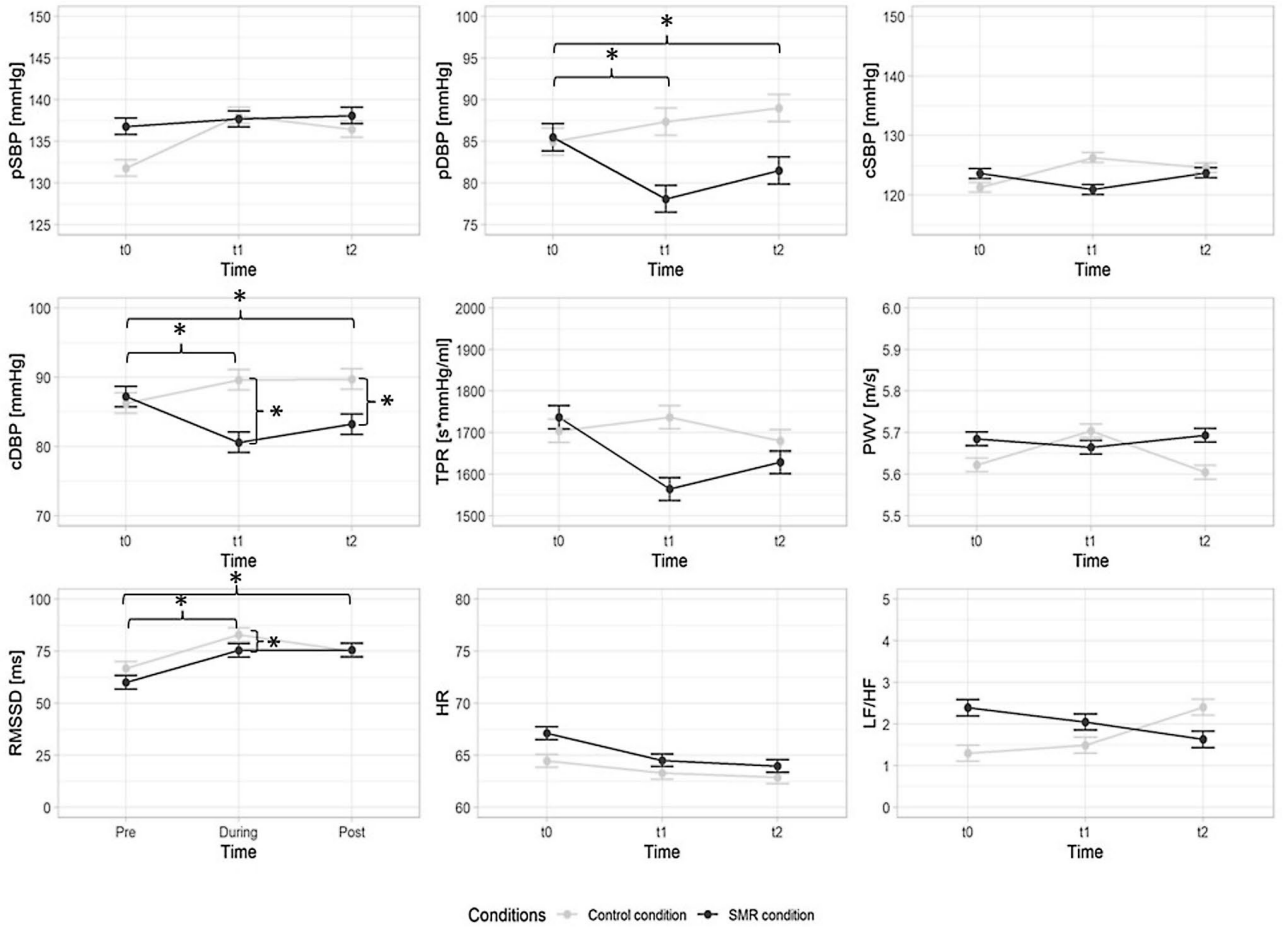
Outcomes during cold pressor test at baseline (t0), 2 min (t1), and 20 min (t2) after the control condition and the self-myofascial release (SMR) condition. Error bars represent standard errors; *pSBP* peripheral systolic blood pressure, *pDBP* peripheral diastolic blood pressure, *cSBP* central systolic blood pressure, *cDBP* central diastolic blood pressure, *TPR* total peripheral resistance, *PWV* pulse wave velocity, *RMSSD* root mean square of successive differences between normal heartbeats, *HR* heart rate, *LF/HF* low-frequency power and high-frequency power quotient

A time × condition interaction was observed for pDBP (*F*(2, 38) = 25.974, *p* < 0.001, *η*^*2*^_*p*_ = 0.578) (Fig. 3). There was a more a more substantial reduction in pDBP observed for the SMR condition from t0 to t1 (*F*(2, 38) = 38.368, *p* < 0.001, *η*^*2*^_*p*_ = 0.669), as well as from t0 to t2 (*F*(2, 38) = 41.530, *p* < 0.001, *η*^*2*^*p* = 0.686) resulting in a lower pDBP at t1 and (*p* < 0.001) t2 (*p* < 0.001). Similarly, a time × condition interaction was observed for cDBP (*F*(2, 38) = 17.018, *p* < 0.001, *η*^*2*^_*p*_ = 0.567), with a stronger reduction following the SMR condition between t0 to t1 (*F*(2,38) = 25.781, *p* < 0.001, *η*^*2*^_*p*_ = 0.665), and t0 to t2 (*F*(2,38) = 28.117, *p* < 0.001, *η*^*2*^_*p*_ = 0.668) resulting in a lower cDBP at t1 and (*p* = 0.002) t2 (*p* = 0.002). Regarding perceived pain, a time × condition interaction was observed (*F*(2,38) = 7.271, *p* = 0.002, *η*^*2*^_*p*_ = 0.277). After the SMR, there was a stronger reduction from t0 to t1 (*F*(2,38) = 9.546, *p* = 0.006, *η*^*2*^_*p*_ = 0.334) compared to the CON condition resulting in significant lower values at t1 (*p* < 0.001). No significant time × condition interaction effects could be observed for pSBP, cSBP, TPR, PWV, HR, RMSSD, and LF/HF (*ps* > 0.05, *η*^*2*^_*p*_ = 0.014–0.146).

## Discussion

This study aimed to evaluate the impact of SMR on cardiovascular parameters at rest and during a standardized stress test. The findings demonstrate that the SMR session effectively lowered hemodynamic parameters both at rest and during the cold pressor test. In addition, perceived pain during the cold pressure test was diminished following the SMR session. Cardiac autonomic function only exhibited improvement after the CON intervention during resting conditions.

### Hemodynamic parameters

Regarding hemodynamic parameters, we observed a significant reduction in pDBP (5 mmHg), cDBP (4 mmHg), and TPR 20 min after the SMR (large effect size), as compared to baseline. These findings are in line with previous research. Lastova et al. (2018) reported a decrease in pDBP of 2 mmHg already 10 min after the SMR, which persisted for up to 30 min after the session. Similarly, in a prior study, our group demonstrated a comparable 2 mmHg reduction in pDBP 30 min following an SMR exercise (Ketelhut et al. 2020). Moreover, Delaney et al. (2002) also noted a significant 3 mmHg decrease in pDBP already 5 min after an upper-body myofascial trigger point massage session. Interestingly no significant reductions in pSBP could be detected in our study, which contradicts the aforementioned studies (Delaney et al. 2002; Lastova et al. 2018; Ketelhut et al. 2020).

Unfortunately, evidence on the effects of SMR on other hemodynamic parameters like central blood pressure, TPR, and PWV is sparse. In a previous study, we could show, that an acute SMR session results in a significant reduction in cSBP, mean arterial pressure, augmentation pressure, and TPR (Ketelhut et al. 2020). A study from Okamoto and colleagues (2013) found significant reductions in brachial-ankle PWV in healthy young adults 30 min after performing SMR of the lower extremities. In our study we only detected a significant reduction with a large effect size in cDBP, and TPR.

Considering our data alongside the previously mentioned literature, it is suggested that acute myofascial therapeutic maneuvers in healthy individuals offer a temporary cardiovascular protective effect by leading to beneficial reductions in hemodynamic parameters. Nevertheless, discrepancies appear to exist among the study outcomes, possibly stemming from variations in study and intervention designs.

### Cardiac autonomic function

In the current study, cardiac autonomic function remained unaffected by the SMR session. It was only following the CON that a decrease in both HR and RMSSD with a large effect size was observed. This contradicts the findings of a prior study conducted by Lastova et al. (2018), which indicated that an acute SMR session led to enhanced parasympathetic activity (measured by HF and RMSSD) and reduced sympathetic activity (measured by LF). The researchers observed a significant increase in RMSSD lasting up to 30 min post the SMR session. In addition, the authors noted a significant decrease in the LF/HF ratio following the SMR session. Another study by Delaney et al. (2002) also reported increased HF at 5 and 20 min after myofascial trigger point massage therapy sessions. Similar to our study Kopec et al. (2022) found no significant effects of a full-body SMR session on RMSSD.

Inconsistencies in results may again be attributed to variations in study design, measurement time points, and the specific SMR intervention utilized. Particularly, the pressure applied during the SMR appears to play a role in modulating responses of cardiac autonomic function. In a study by Diego et al. (2009), a more intense pressure massage yielded a parasympathetic nervous system response, while a lower pressure massage elicited a sympathetic nervous system response. The authors suggested that only the more intense pressure stimulated dermal pressure receptors, subsequently leading to increased vagal efferent activity. Regrettably, previous studies that reported positive effects on cardiac autonomic function did not report pressure intensity during the SMR session. Besides pressure intensity, various protocols involving different techniques and exercises, as well as targeting different muscle groups, could contribute to conflicting results. Furthermore, our SMR protocol might have been more physically demanding than the passive myofascial trigger point massage therapy applied in the study by Delaney et al. (2002), leading to a stronger and more sustained activation of the sympathetic nervous system. Our SMR protocol might even be more physically demanding compared to the one applied by Lastova et al. (2018), which included rolling the lower and upper back, engaging less muscle groups to offset weight and ensure stability.

### Stress response

Regarding the stress response during the cold pressor test, our findings indicate that SMR mitigated the rise in both pDBP and cDBP for up to 20 min after the session. Unfortunately, no previous study has evaluated blood pressure responses during the cold pressor test following an SMR session. Nevertheless, previous research has demonstrated that various types of exercise lead to a similar reduction in hemodynamic responses during a cold pressor test (Ebbesen et al. 1992; Milatz et al. 2015; Ketelhut et al. 2016, 2022).

In addition, participants reported experiencing lower perceived pain during the cold pressor test following the SMR session. Unfortunately, no prior study has assessed the cardiovascular stress response or pain perception during a cold pressor test after SMR. However, a study by Cheatham and Baker (2017) demonstrated that an SMR intervention led to an immediate increase in pressure pain threshold. This finding aligns with Aboodarda et al. (2015), who also noted an increase in pressure pain threshold after a rolling massage. A study conducted by Cavanaugh et al. (2017) found that an acute bout of SMR diminished the testing-induced increase in pain perception associated with submaximal tetanic evoked stimulation.

It appears that mechanical stimuli from SMR result in a reduction in pain perception and decreases cardiovascular reactivity. The fact that frequent fluctuations in blood pressure have been proposed to directly influence the vascular system, potentially leading to damage and impacting arterial compliance, which, in the long term could contribute to hypertension (Folkow 1993), underscores the significance of these results.

### Mechanisms

The mechanisms underlying the beneficial effects of SMR on cardiovascular parameters are complex and not fully understood. However, it can be speculated that the mechanical stress applied to the vessels and tissue during SMR triggers the production of nitric oxide (NO). Studies on rats have demonstrated that a stretching stimulus applied to vessels increased NO production from vascular endothelial cells (Kuebler et al. 2003). Similar findings were observed in bovine vascular endothelial cells subjected to cyclic stretch stimuli, showing upregulation of endothelial NO synthase mRNA expression (Awolesi et al. 1995). Furthermore, it has been reported that also circumferential deformation of vessels leads to endothelial NO production (Lu and Kassab 2015). Therefore, the stretching and pressure stimulus induced by SMR in the present study may have trigger NO-related vasodilation and subsequently lead to a decrease in pDBP, cDBP, and TPR. Unfortunately, NO was not assessed in this study, however, Okamoto et al. (2013) reported an increase in circulating NO levels after an acute SMR session.

Interestingly, SMR did not yield changes in pSBP, cSBP, and HRV parameters. This could potentially be explained by the fact that the SMR session led to autonomic nervous system activation. During SMR, the participants achieved a mean HR of 96 ± 14 min^−1^, while during the CON session, the mean HR was 64 ± 10 min^−1^. It can be speculated that this activation of the sympathetic nervous system, along with a subsequent increase in cardiac output, attenuated a potential decrease in pSBP caused by vasodilation. According to the HRV parameters, sympathetic nervous system activation remained elevated 20 min post the SMR session, thereby ensuring a stable pSBP regardless of the reduction in TPR. Therefore, drawing from the current results, it can be concluded that during the initial 20 min after an SMR session, the alterations in vascular tone and blood flow regulation induced by mechanical pressure might constitute the principal mechanism driving the hemodynamic changes, rather than its impact on cardiac autonomic function.

Regarding the stress response, previous studies have reported that beneficial effects of exercise on cardiovascular reactivity during the cold pressor test may be attributed to a reduction in perceived pain, which is considered the primary trigger of the cardiovascular response during this test (Saccò et al. 2013). In the present study, participants reported experiencing lower perceived pain during the cold pressor test following the SMR session. Research suggests that neurophysiological responses following SMR may lead to pain reduction through the activation of descending inhibitory neural pathways (Jay et al. 2014), and decreased nociceptor activation (Macdonald et al. 2014; Aboodarda et al. 2015). However, regardless of the identified analgesic effect of SMR, no influence on HRV parameters during the cold pressure test was observed, contradicting this presumption. Therefore, it can be hypothesized that the decrease in TPR resulting from SMR could be accountable for the attenuated stress response observed after the intervention.

### Practical implications

The scientific evidence supporting SMR as a method to improve hemodynamic parameters holds substantial practical implications for both healthcare professionals and individuals seeking non-pharmacological interventions for cardiovascular health. If these acute responses can result in long-term adaptations, SMR offers a valuable, non-invasive, and easily accessible intervention as an adjunct to conventional treatments for hypertension and cardiovascular management. Healthcare professionals, fitness trainers, and individuals can leverage SMR techniques to promote overall cardiovascular well-being.

One notable advantage of SMR is its potential to offer a promising alternative to aerobic exercise, especially for individuals with limitations in engaging in aerobic exercises for various reasons. SMR elicit a lower metabolic demand than aerobic exercise, making it particularly interesting for those with reduced exercise capacity or mobility challenges.

Despite these practical implications, further research should explore the long-term effects and optimal protocols of SMR in different populations. Understanding how SMR can be tailored to specific individuals will be crucial in maximizing its benefits and ensuring safety.

### Limitations

Several limitations need to be acknowledged that may influence the interpretation of the study results. First, the study focused on healthy individuals with specific inclusion criteria, restricting the generalizability of the results to broader populations, such as individuals with underlying medical conditions or those on cardiovascular medications. Future studies should consider including a more diverse range of participants to better reflect real-world scenarios. Second, only the acute effects of an SMR were assessed. This limits the ability to draw definitive conclusions about the sustained effects of SMR on cardiovascular health over an extended period. However, exercise research corroborates the theory that the lasting positive influence on hemodynamics resulting from engaging in regular exercise programs primarily arises from the cumulative effects of the changes observed after each individual exercise session (Whyte et al. 2010). Third, despite efforts to control for confounding factors, the possibility of uncontrolled variables influencing the results cannot be completely ruled out. Factors such as dietary habits, hydration status, and sleep quality were not measured or controlled for in the study. Fourth, the study employed a specific SMR protocol targeting particular muscle groups. Different SMR protocols or variations in duration may yield diverse outcomes. Fifth, the postintervention period was limited to 20 min, which might not have allowed sufficient time for complete recovery in some HRV variables and blood pressure. Extending the postintervention observation period could provide more comprehensive insights into the short-term effects of SMR.

## Conclusion

The findings from this study indicate that a single session of SMR provides positive cardiovascular effects at rest and during a standardized stress test in healthy normotensive individuals. It is postulated that SMR exerts mechanical stress on the vascular system similar to massage therapy and stretching exercises, leading to temporary improvements in hemodynamic parameters. Future research should explore the possibility of repeated exposure to these acute responses leading to long-term adaptations akin to regular, moderate aerobic exercise, massage therapy, and static stretching. If regular SMR demonstrates a lasting reduction in arterial pressure and stiffness, it could be considered as a valuable addition to physical activity programs aimed at promoting health and reducing the burden of cardiovascular disease.

## Data Availability

All raw data are available on request to the corresponding author.

## Abbreviations

ANOVA: Analysis of variance
BMI: Body Mass Index
cDBP: Central diastolic blood pressure
CON: Control condition
cSBP: Central systolic blood pressure
Fig.: Figure
HR: Heart rate
HRV: Heart rate variability
LF/HF: Quotient of low-frequency power and high-frequency power
NO: Nitric oxide
η2p: Partial eta-squared
pDBP: Peripheral central blood pressure
pSBP: Peripheral systolic blood pressure
PWV: Pulse wave velocity
RMSSD: Root mean square of successive differences between normal heartbeats
SMR: Self-myofascial release
TPR: Total peripheral resistance
WHtR: Waist-to-height ratio
